# Validation and improvement of the mental health literacy scale to assess undergraduate nursing students in South Africa

**DOI:** 10.4102/sajpsychiatry.v32i0.2533

**Published:** 2026-01-28

**Authors:** Lauren Blommetjies, Elmari Deacon

**Affiliations:** 1 COMPRES, Faculty of Health Sciences, North-West University, Potchefstroom, South Africa; 2 Optentia Research Unit, Faculty of Humanities, North-West University, Potchefstroom, South Africa

**Keywords:** mental health literacy scale, nursing students, primary healthcare, South Africa, Psychometric properties

## Abstract

**Background:**

Mental Health Literacy (MHL) refers to knowledge, skills and attitudes related to mental health and is often measured by the revised Mental Health Literacy Scale (MHLS). This scale has been validated on a group of primary healthcare (PHC) workers in Zambia and South Africa, but its usefulness for a group of nursing students has not been investigated until now.

**Aim:**

To investigate the psychometric properties of the revised scale for a group of undergraduate nursing students at a South African university, while also assessing the level of MHL in the group under investigation.

**Setting:**

Nursing students (*N* = 121) studying for their Bachelor of Nursing degree at a South African public university.

**Methods:**

A quantitative, cross-sectional survey was conducted between July 2023 and August 2023, using a purposive sampling technique. A socio-demographic data form and a revised version of the MHLS were used to collect data online. Descriptive statistics, Cronbach’s alpha, exploratory factor analysis (EFA) and confirmatory factor analysis (CFA) were used to analyse the data.

**Results:**

The EFA and CFA seemed to confirm a new 5-factor structure, including only 17 items. This structure holds similar factors to the MHL subscales. The reliability of most of the new factors was good, with two factors that need further intervention. This version of the MHLS proposed satisfactory levels of MHL for this group.

**Conclusion:**

This study proposes a shortened, 5-factor structure version of the MHLS to be used to assess nursing students.

**Contribution:**

The revised 5-factor structure measure can potentially identify gaps in the MHL of students, and this can inform appropriate interventions to address the MHL needs in South Africa generally.

## Introduction

The term mental health literacy (MHL) was coined in the late 1990s by Anthony Jorm, and since then, it has been extensively researched in Australia and other developed countries.^1^ Literacy in this context entails seven features: the capacity to recognise mental disorders; how to seek mental health information; awareness of mental health risk factors; appreciation of the aetiology of mental disorders; knowledge of self-treatment; knowledge of professional help available; and attitudes that promote recognition of appropriate health-seeking behaviour.^2^ While the emerging MHL literature shows much promise for the mental health field, there are several challenges, especially regarding the measurement of MHL.

Most MHL measurements fail to simultaneously account for all the features of MHL and describe adequate psychometric properties.^3,4^ Another challenge is the unknown status of the significant contextual contributions to MHL, such as culture, ethnicity, geographic location, as well as the educational background of its practitioners and their health status.^5^ As a remedy to address these unknowns, recent studies have attempted to bridge the gaps by developing strong psychometric measures that quantify MHL, assess its many components and target specific populations such as healthcare workers.

O’Connor and Casey^4^ developed the Mental Health Literacy Scale (MHLS), the first scale-based measure to assess all attributes of MHL that were found to have acceptable psychometric properties and can be easily administered and scored. Although MHL comprises the seven features mentioned above, O’Connor and Casey^4^ developed their scale to measure only six attributes, as feedback from a clinical panel suggested that, generally, there is a lack of knowledge to distinguish between risk factors for mental illness and its causes. These traits have therefore been integrated into one: knowledge of risk factors and causes. Although the MHLS was recently revised and content-validated in South Africa and Zambia by a diverse expert panel comprising primary healthcare (PHC) staff and research professionals,^6^ it has not been administered to a group of nursing students to assess its psychometric properties, as reported here.

As the PHC nurse is often the first contact with a patient, it is imperative that these healthcare workers have sufficient command of MHL. Unfortunately, Lahti and colleagues^7^ found that these workers’ recognition and screening of mental health problems at the PHC level is poor. Indeed, Dirwayi^8^ reported that the majority of nurses showed subtle negative attitudes toward people with mental illness. To improve the MHL of nurses, therefore, we need to be able to measure these attributes accurately. If we can identify deficits in MHL in nursing students early, we can adopt curricula in their training to empower them to fulfil this need; hence, the study reported here investigated the viability of using the revised MHLS scale in a group of nursing students in South Africa.

To date, there has been no investigation of the level of MHL among South African nursing students. Indeed, there have been only two studies that have validated the MHLS in a South African context,^6,9^ thereby necessitating the need for its critical review in current circumstances. The aim of our study was to investigate the psychometric properties of the revised MHLS in a group of undergraduate nursing students and to assess the level of MHL in the group under investigation.

## Research methods and design

### Study design

A quantitative, cross-sectional design was used to investigate the psychometric properties of the MHLS in the context of nursing students and determine their levels of MHL.^9^

### Setting, study population and sampling strategy

The study was conducted at the North-West University, South Africa, on a population of 121 nursing students in their first to fourth years towards completing the Bachelor of Nursing degree. We employed a purposive sampling technique to include students from all 4 years as the study was concluded in the first semester, before the fourth-year students completed the module on mental health. Students who completed their fourth year already, were excluded, as they had completed the module on mental health.

### Data collection

The data collection tool used was the revised version of the MHLS,^9^ which consists of six subscales, addressing: recognition of disorders; knowledge of how to seek mental health information; knowledge of risk factors and causes; knowledge of self-treatments; knowledge of professional help available; and attitudes that promote recognition and appropriate help-seeking. The scale uses a Likert response scale (items 1 to 15: 1 – very unlikely to 4 – very likely; items 16 to 28: 1 – strongly disagree to 5 – strongly agree; items 29 to 35: 1 – definitely unwilling to 5 – definitely willing). The difference in the ranges of the Likert scales does not influence the analysis when using Mplus,^10^ and therefore rescaling is unnecessary. Although the original MHLS was found to have good reliability (*r*[69] = 0.797, *p* < 0.0014), Korhonen and colleagues found Chronbach’s alpha values for three of the subscales to be below the acceptable level, but the reliability for the combined scale was acceptable (0.804). Korhonen and colleagues^6,9^ found that the MHLS has sufficient validity in the South African context items loading according to the seven features of MHL.

### Procedure

Students were invited to participate in the study by means of a link to a learning management system. Those interested had to click on the link for more information on the project as well as the consenting process. After completing the latter, participants were allowed to complete the questionnaire online. As reimbursement for the time they spent completing the form, they could provide their email address to receive a R30 coffee voucher. Data were collected between July and August 2023.

### Data analysis

The quantitative data analysis was conducted using IBM^®^ Statistical Package for the Social Sciences (SPSS) Version 28 software,^11^ as well as Mplus, 8.10.^10^ Descriptive statistics were used to analyse participant information, as well as to determine possible skewness and kurtosis. Both total and subtotal scores were calculated between scale items. Cronbach’s alpha and Omega were used to report reliability, while exploratory factor analysis (EFA), including both principal component analysis (PCA) and principal axis factoring (PAF) was used to investigate possible different factor structures for the measure. A CFA was run only to check the possible model fit.

### Ethical considerations

Ethical clearance to conduct this study was obtained from the North-West University Health Research Ethics Committee (No. NWU-00188-22-A1). Online, formal, written informed consent was obtained from all participants, who were provided with numbers to ensure confidentiality; no identifying details were shared.

## Results

A total of 121 student nurses, whose characteristics are reported in Table 1, completed the online questionnaire, with no questionnaires excluded. The gender distribution of the participants was as follows: 92% (*n* = 111) were female, and 8% (*n* = 10) were male. Most of the participants (69%) were between the ages of 20 and 24. In terms of home language, 44% (*n* = 53) listed Afrikaans and 36% (*n* = 44) gave their home language as Setswana. Most of the participants (33%) were in their fourth year, followed by those in their first year (27%), their third year (21%) or their second year (19%). Over 82% (*n* = 100) of the students reported having less than 1 year of professional nursing experience; the remaining 17% (*n* = 21) reported a corresponding 1–5 years.

**TABLE 1:** Characteristics of the participants (*N* = 121).

Item	Category	Frequency	%
Gender	Male	10	8.3
	Female	111	91.7
Age (years)	< 20	35	29.0
	20–24	83	68.5
	> 24	3	2.4
Home language	English	5	4.1
	Afrikaans	53	43.8
	Setswana	44	36.4
	isiZulu	3	2.5
	isiXhosa	1	0.8
	Sepedi	6	5.0
	Sesotho	4	3.3
	Shona	2	1.7
	Tshivenda	1	0.8
	English and Afrikaans	2	1.7
Year of study	1st	33	27.3
	2nd	23	19.0
	3rd	25	20.6
	4th	40	33.1
Years of experience	< 1	100	82.6
	1–5	21	17.4

### Psychometric properties of the revised mental health literacy scale

The data were found to be non-normally distributed, showing characteristics of skewness and kurtosis. When dividing the skewness and kurtosis statistics by their relevant standard error, values between −1.96 and +1.96 are considered normal. In this sample (including demographic information and all 35 items), 77.5% indicated skewness and 50.0% kurtosis. Therefore, the robust maximum likelihood (MLR) estimator for the analyses in Mplus 8.10 was used.^10^ A confirmatory factor analysis (CFA) for the MHLS was specified in Mplus 8.10^10^ according to the original 6-factor solution; however, as this factor structure was not supported in our sample (see Table 2), an EFA (with PCA and PAF) was performed, also in Mplus, 8.10,^10^ which included all the items. To approximate the possible number of underlying factors, eigenvalues were used, with values above 1.00 indicating that a certain number of items might have enough in common to represent one factor. According to the eigenvalues, up to 12 factors were possible (PCA), although the PAF results indicated only 7, as the possible solutions with 8 to 12 factors did not converge, thus indicating issues such as overfactoring, a small sample size or extreme multicollinearity.

**TABLE 2:** Fit statistics of confirmatory factor analyses from exploratory factor analysis results.

Model	χ^2^	*df*	MLR-adjusted χ^2^	RMSEA	CFI	TLI	SRMR
2-factor	149.93	89	160.29	0.08	0.88	0.86	0.07
3-factor	202.53	116	211.87	0.08	0.84	0.82	0.08
4-factor	65.82	34	70.51	0.09	0.91	0.88	0.06
5-factor	197.65	142	202.93	0.06	0.90	0.88	0.07
6-factor	396.06	284	383.34	0.06	0.85	0.83	0.09
7-factor	-	-	-	-	-	-	-

Note: The residual covariance matrix is not positive definite for the 7-factor model.

χ^2^, Chi squared; *df*, degrees of freedom; MLR-adjusted χ^2^, maximum likelihood robust adjusted χ^2^; RMSEA, root mean square error of approximation; CFI, comparative fit index; TLI, Tucker–Lewis index; SRMR, standardised root mean square residual.

Each of the possible factor structures, specified according to significant loadings identified in the respective PAFs, was tested in Mplus – the fit statistics from the first run of each factor structure are reported in Table 2. Owing to the small sample size, the data could not be split into two different sets, as per good practice guidelines, in order to perform any CFA in a separate data set. However, to be able to propose a factor structure for future study, every possible factor structure from the EFA was inspected and/or developed to determine the best fit through CFA for further analysis. The 2-factor structure decreased in fit, while the 3-factor structure improved (although not to acceptable levels), and the 4-factor structure could not be developed any further. The 1-factor and 6-factor structures were abandoned as too many items with low factor loadings would have had to be removed, but the 5-factor structure achieved acceptable fit after the removal of two items with factor loadings below 0.35 (χ^*2*^ [*df*] = 142.30 [109]; MLR-adjusted χ^*2*^ = 148.33; RMSEA = 0.05; Comparative Fit Index [CFI]/Tucker-Lewis Index [TLI] = 0.94/0.92; standardised root mean square residual [SRMR] = 0.06).

The descriptive statistics of the proposed factor structures, Cronbach’s alpha and Omega reliability coefficients, and evaluated relationships are presented in Table 3. The first factor in the revised structure represented items from the first subscale: Recognition of mental illness (items 4, 5 and 7; measured on a scale of 1–4), whereas the second factor consisted of only two items (12 and 28; measured on a scale of 1–4 and 1–5, respectively), both relating to the treatment of mental illness. The third factor in the revised structure combined items from two subscales: Knowledge of how to seek information (items 16, 17 and 19; measured on a scale of 1–5) and two items from attitudes (25 and 26; measured on a scale of 1–5), hence this factor was named mental health information-seeking and sharing, as some items also relate to sharing diagnosis. The fourth factor identified in the new factor structure consists of items 29, 30, 31 and 32 (measured on a scale of 1–5). All of these items belong to the original subscale attitudes. In the revised structure, this subscale is also represented in factor 5 by items 33, 34 and 35 (measured on a scale of 1–5). On closer investigation, the difference between these factors was that the first is concerned with mental health attitudes regarding significant others on a micro level (named mental health attitude–micro level), whereas the fifth factor was concerned with mental health attitudes of others in the larger community a person lives in, or macro level (named mental health attitude–macro level).

**TABLE 3:** Descriptive statistics, reliability coefficients and correlations.

Variable		Mean	s.d.	α	Ω	1	2	3	4
1.	Recognition of mental illness	3.27	0.65	0.56	0.56	-	-	-	-
2.	Treatment of mental illness	2.06	0.59	−0.74	-	−0.44†**	-	-	-
3.	Mental health information-seeking and sharing	3.21	0.45	0.10	-	0.09	0.21	-	-
4.	Mental health attitudes – Micro	3.86	0.82	0.87	0.87	0.12	0.25	0.36†**	-
5.	Mental health attitudes – Macro	3.45	0.93	0.79	0.80	0.20	0.28*	0.29*	0.75‡**

s.d., standard deviation.

* , *p* < 0.05;

** , *p* < 0.01.

† , *r* > 0.30;

‡ , *r* > 0.50.

The internal consistencies (Cronbach’s alpha and Omega) of mental health information-seeking and sharing, mental health attitude–micro level and mental health attitude–macro level were all acceptable at α = 0.75 and *ω* = 0.75, α = 0.87 and *ω* = 0.87, and α = 0.79 and *ω* = 0.80, respectively. The internal consistency of recognition of mental illness was very low (α = 0.56 and *ω* = 0.56), and this needs to be further investigated. The treatment of mental illnesses’ alpha value was above the cut-off point, but remained negative, regardless of the reversal of negative items. It might be an issue that there are only two items for the factor; it could be because of the small sample size, or a negative inter-item correlation, but in this case, it is probably because the two items were measured on different Likert scales (item 12 on 1–4 and item 28 on 1–5). The internal consistency for the complete measure, consisting of 17 items, was α = 0.77 and *ω* = 0.83; its mean overall was 3.70 with a standard deviation (s.d.) of 0.45.

## Discussion

This study set out to investigate the usefulness of the MHLS in a group of nursing students in South Africa.

While Korhonen and colleagues^9^ reported that the revised version of the scale has a valid structure with strong internal consistency, our results do not support this finding. The identification of a 5-factor model, consisting of 17 items, provides a potentially more appropriate framework for understanding MHL in this context. These factors represent key domains, such as attitudes at the individual and societal level, information behaviour and recognition and treatment of mental illness.

The internal consistencies for mental health attitudes on a micro level, mental health information-seeking and sharing, and mental health attitudes on a macro level were all high; however, the internal consistencies for recognition of mental illness and treatment of mental illness were found to be very low and negative, respectively. It should be noted that Korhonen and colleagues^9^ had a total of 343 participants in their study, whereas our study had a total of 121 participants. Furthermore, Korhonen and colleagues^9^ used PCA, whereas we used not only EFA, including PCA, but also PAF, as well as CFA. Recurrent findings have indicated that PCA inflates factor loadings, changing the factor structure as a result, especially when there are few variables.^12,13^ A PCA helps identify the possible number of underlying factors in a data set, whereas a PAF evaluates the item loadings on the number of factors specified (in this case, an oblique Geomin rotation was used). Neither of the two provides an actual model or specific fit statistics. In Mplus, the two steps are combined to provide all the necessary information in one step. From the EFA results, a CFA indicates whether a specifically specified model fits the data, what the relationships between factors look like (and if they exist), and where possible issues with the model or data could be. This step is not possible in SPSS, and AMOS can take long, intensive work; thus, Mplus is ideal for the CFA process.

Preliminary findings using the revised 5-factor model, as presented in this study, indicate that the sample of undergraduate nursing students had a satisfactory level of MHL. Crisp and colleagues^12^ proposed that this may have been influenced by their greater exposure to university education in general, a conclusion that has been confirmed by Furnham and Hamid,^13^ who also found that education had a positive effect on MHL. The lower scores obtained specifically pertaining to information-seeking and treatment of mental illness are consistent with the literature; these nurses did not consider themselves to be sufficiently prepared to work with people with mental illnesses.^14,15,16^ According to Sand-Jecklin,^17^ incorporating MHL education and skills training into nursing and health curricula would allow students to evaluate patients’ MHL and offer interventions or support to make sure the patients understand the value of health information and make the most effective use of their resources. These findings should be interpreted with some caution, though, as, because of the small sample size, the CFA had to be conducted on the same data as the EFA.

Although this was a small cross-sectional study, with a low response rate, which limited the generalisability of the results, its contribution in terms of the development of the MHLS to be useful in the education sector is clear. For future use, adjusting the Likert scales to use the same range throughout the questionnaire (e.g. by removing the neutral option from items 16–35) could strengthen the reliability of all the factors, especially the mental health treatment factor. Future research on the scale should focus on adapting or removing or adding applicable items through consultation with experts to improve content validity, and further clarifying the factor structure by conducting a larger-scale study, using EFA and CFA to investigate the validity of the structure.

## Conclusion

Increasing the MHL of healthcare students could assist in integrating mental health treatment into PHC services, particularly in low- and middle-income countries.^18^ This study has shown that the revised MHLS is not valid for a group of nursing students and requires more research to confirm the 5-factor structure and improve its reliability. Furthermore, we propose the use of CFA in future studies to explain relationships between variables and to analyse factor structures.

## References

[1] Tay JL, Tay YF, Klainin-Yobas P. Mental health literacy levels. Arch Psychiatr Nurs. 2018;32(5):757–763. 10.1016/j.apnu.2018.04.007 30201205

[2] Jorm AF, Korten AE, Jacomb PA, Christensen H, Rodgers B, Pollitt P. ‘Mental health literacy’: A survey of the public’s ability to recognise mental disorders and their beliefs about the effectiveness of treatment. Med J Aust. 1997;166(4):182–186. 10.5694/j.1326-5377.1997.tb140071.x 9066546

[3] Kutcher S, Bagnell A, Wei Y. Mental health literacy in Secondary Schools. Child Adolesc Psychiatr Clin North Am. 2015;24(2):233–244. 10.1016/j.chc.2014.11.007 25773321

[4] O’Connor M, Casey L. The Mental Health Literacy Scale (MHLS): A new scale-based measure of mental health literacy. Psychiatr Res. 2015;229(1–2):511–516. 10.1016/j.psychres.2015.05.064 26228163

[5] Wei Y, McGrath PJ, Hayden J, Kutcher S. Mental health literacy measures evaluating knowledge, attitudes and help-seeking: A scoping review. BMC Psychiatry. 2015;15(1):1–20. 10.1186/s12888-015-0681-9 26576680 PMC4650294

[6] Korhonen J, Axelin A, Grobler G, Lahti M. Content validation of Mental Health Literacy Scale (MHLS) for primary health care workers in South Africa and Zambia – A heterogeneous expert panel method. Glob Health Action. 2019;12(1):1668215. 10.1080/16549716.2019.1668215 31699016 PMC6853208

[7] Lahti M, Groen G, Mwape L, et al. Design and development process of a youth depression screening m-Health application for primary health care workers in South Africa and Zambia: An overview of the MEGA project. Issues Ment Health Nurs. 2019;41(1):24–30. 10.1080/01612840.2019.1604919 31225763

[8] Dirwayi NP. Mental illness in primary health care: a study to investigate nurse’s knowledge of mental illness and attitudes of nurses toward the mentally ill. (Thesis). University of Cape Town, Faculty of Humanities, Department of Psychology. 2002. Available from: http://hdl.handle.net/11427/7930

[9] Korhonen J, Axelin A, Katajisto J, Lahti M. Construct validity and internal consistency of the revised mental health literacy scale in South African and Zambian contexts. Nurs Open. 2021;9(2):966–977. 10.1002/nop2.1132 34822738 PMC8859090

[10] Stockemer D. Quantitative methods for the social sciences: A practical introduction with examples in SPSS and Stata. Cham: Springer International Publishing; 2019.

[11] IBM Corp. IBM SPSS statistics for Windows, Version 28.0. IBM Corp; 2023, Armonk, NY, US.

[12] Crisp A, Gelder M, Goddard E, Meltzer H. Stigmatization of people with mental illnesses: A follow-up study within the changing minds campaign of the Royal College of Psychiatrists. World Psychiatry. 2005;4(2):106–113. 16633526 PMC1414750

[13] Furnham A, Hamid A. Mental health literacy in non-western countries: A review of the recent literature. Ment Health Rev J. 2014;19(2):84–98. 10.1108/MHRJ-01-2013-0004

[14] Saito AS, Creedy DK. Determining mental health literacy of undergraduate nursing students to inform learning and teaching strategies. Int J Ment Health Nurs. 2021;30(5):1117–1126. 10.1111/inm.12862 33760328

[15] Weare R, Green C, Olasoji M, Plummer V. ICU nurses feel unprepared to care for patients with mental illness: A survey of nurses’ attitudes, knowledge, and skills. Intensive Crit Care Nurs. 2019;53:37–42. 10.1016/j.iccn.2019.03.001 30878535

[16] Kemp CG, Mntambo N, Weiner BJ, et al. Pushing the bench: A mixed methods study of barriers to and facilitators of identification and referral into depression care by professional nurses in KwaZulu-Natal, South Africa. SSM Ment Health. 2021;1:100009. 10.1016/j.ssmmh.2021.100009 34541564 PMC8443051

[17] Sand-Jecklin K, Murray B, Summers B, Watson J. Educating nursing students about health literacy: From the classroom to the patient bedside. Online J Issues Nurs. 2010;15(3). 10.3912/OJIN.Vol15No03PPT02

[18] Marangu E, et al. Assessing Mental Health Literacy of Care Workers in Kenya: A Cross Sectional Survey. International Journal of Mental Health Systems. 2021;15(55). 10.1186/s13033-021-00481-z PMC8170792 34074318

